# Immune-Enhancement Effects of 6-Methoxykaempferol on Cyclophosphamide-Induced Immunosuppression via Improving Antioxidant Enzyme Expression, NF-κB and MAPK Signaling, and Modulating Gut Microbiome

**DOI:** 10.3390/antiox15030334

**Published:** 2026-03-06

**Authors:** Na-Yeon Jang, Varun Jaiswal, Miey Park, Hae-Jeung Lee

**Affiliations:** 1 Department of Food and Nutrition, College of BioNano Technology, Gachon University, Seongnam-si 13120, Republic of Korea; nayeon6857@naver.com (N.-Y.J.); mpark@gachon.ac.kr (M.P.); 2 Institute for Aging and Clinical Nutrition Research, Gachon University, Seongnam-si 13120, Republic of Korea; 3 Department of Health Sciences and Technology, Gachon Advanced Institute for Health Sciences and Technology (GAIHST), Gachon University, Incheon 21999, Republic of Korea

**Keywords:** 6-methoxykaempferol, immune-enhancement, functional food, microbiome, flavonol

## Abstract

The immune system maintains homeostasis through coordinated innate and adaptive responses, and its imbalance increases disease susceptibility. The immunomodulatory effects of 6-methoxykaempferol (6MK), a methoxylated flavonoid found in sweet cherries, were studied in a mouse model of cyclophosphamide (CPA)-induced immunosuppression. The expression of key signaling proteins in the NF-κB and MAPK pathways was studied to explore the underlying molecular mechanisms. The Toll-like receptor-4/myeloid differentiation factor-2 receptor complex (TLR4/MD2), which can stimulate the immune response by activating these pathways, was used to study possible interactions with 6MK using docking analysis. 6MK administration significantly restored immune organ integrity (spleen up to 15.1% and thymus up to 16.8%), enhanced NK cell function (up to 43.8%), promoted T (up to 24.5%) and B cell proliferation (up to 26.4%), increased pro- and anti-inflammatory cytokine (IL-1β, IL-6, TNF-α, IL-4, IL-10, and TGF-β) levels, and elevated NO (up to 25.6%) and immunoglobulin (IgG, IgA, and IgM) concentrations. Additionally, 6MK upregulated antioxidant enzymes (CAT, HO-1, and SOD) and reactivated suppressed NF-κB and MAPK pathways. The docking-supported hypothesis, based on putative interactions and the estimated free energy of binding, suggests that 6MK possesses agonistic potential for the TLR4/MD2. Changes in the gut microbiome due to 6MK treatment, such as an increase in alpha diversity, abundance of *Dorea longicatena*, and the upregulation of formaldehyde-consuming pathways, may also contribute to immune enhancement. These findings show that 6MK may alleviate immunosuppression, suggesting its potential for future studies targeting immune-related diseases and conditions.

## 1. Introduction

Immune strengthening is an important aspect of various functional foods that contributes to their health-promoting effects. The immune system protects organisms from infectious and serious non-infectious diseases and conditions, including allergies, inflammation, lymphoid hyperplasia, granulomatous disease, liver diseases, progressive lung disease, autoimmune diseases, and cancer [1,2]. The immune system is conventionally divided into two interrelated components: the innate immune system, which provides immediate but nonspecific defense, and the adaptive immune system, which offers delayed, but specific responses to pathogens [3]. Immune dysregulation impairs host defense mechanisms, predisposing individuals to an increased susceptibility to a broad spectrum of diseases. Several avoidable and unavoidable factors can suppress the functions of the immune system, such as malnutrition, age, chemotherapy, and immune system-related diseases. In the present scenario, the increased burden of lifestyle factors such as junk food, physical inactivity, psychosocial stress, and exposure to xenobiotic compounds can also contribute to poor immunity [4,5,6,7]. A healthy diet rich in phytochemicals such as flavonoids can strengthen the immune system by targeting crucial targets associated with immunity [8]. 3,5,7,4′-Tetrahydroxy-6-methoxyflavone, also known as 6-methoxykaempferol (6MK), is a flavonol reported in various fruits and plants such as *Flaveria trinervia* [9], *Tetragonia tetragonoides* [10], *Tephrosia purpurea* [11], and different species of *Centaurea* [12].

6MK is expected to be a biologically active flavonol owing to its similarity to fisetin, kaempferol, and galangin, which have multiple pharmacological activities [13,14]. However, few studies have analyzed the biological activity of 6MK. In a preliminary study, the non-enzymatic antioxidant activity of 6MK was observed using 2,2-diphenyl-1-picrylhydrazyl (DPPH) and 2,2′-azinobis(3-ethylbenzothiazoline-6-sulfonic acid) (ABTS) assays [10]. In a screening study of p38 mitogen-activated protein kinase (MAPK) and c-Jun N-terminal kinase (JNK) 3, 42 naturally occurring flavonoids, including 6MK, were studied [15]. However, these preliminary in vitro studies did not focus on 6MK, and there is no evidence of its physiological relevance in vivo conditions. The immune-enhancing effects of phytochemicals, especially flavonols, are well known and can be a major contributing factor to their multiple pharmacological properties against critical diseases [14,16,17]. Notably, the immunomodulatory effects of 6MK have not been investigated in vivo.

Thus, this study aimed to investigate the immune-enhancing activity of 6MK using a cyclophosphamide (CPA)-induced immunosuppressive mouse model. CPA is a widely used alkylating agent with antineoplastic and immunosuppressive properties [18,19]. During metabolism, CPA generates excessive reactive oxygen species (ROS), leading to oxidative stress and subsequent damage to DNA, proteins, lipids, and other cellular structures [20]. Owing to its profound immunosuppressive effects, CPA is frequently used to establish immunocompromised murine models for immunological and pharmacological research. Nuclear factor-κB (NF-κB) and MAPK signaling pathways constitute the two principal regulatory cascades in immune modulation. Activation of key proteins in these pathways was analyzed to study the molecular mechanisms involved in immune modulation by 6MK. Activation of the Toll-like receptor-4/myeloid differentiation factor-2 receptor complex (TLR4/MD2) can stimulate immune responses via these pathways [21,22]. Thus, the binding of 6MK to TLR4/MD2, which may activate TLR4/MD2, was evaluated using molecular docking analysis [21,22]. Molecular docking is a powerful in silico method that aids in understanding drug mechanisms in a cost- and time-effective manner. Recent studies have highlighted its utility in investigating the molecular targets of natural compounds, such as nutraceuticals [23]. In the case of TLR4/MD2, the availability of a crystal structure with a well-defined binding pocket ensures the feasibility and reliability of molecular docking, making it the preferred choice for this analysis [24].

The prebiotic function of similar flavonols, such as galangin, quercetin, and fisetin, in increasing the abundance of health-promoting probiotic bacteria, such as *Bifidobacterium adolescentis*, has been reported [25]. Phytochemicals that positively modulate the gut microbiome may exert health-promoting effects, including immune enhancement [26]. The gut–immune axis is a bidirectional communication network linking the gut microbiota, the intestinal barrier, and the systemic immune system. This axis plays a significant role in immunomodulation, primarily mediated by gut microbiome metabolites such as short-chain fatty acids (SCFAs) and tryptophan derivatives [27,28,29]. Therefore, this study investigated the effect of 6MK on the gut microbiome to assess its prebiotic potential of 6MK, which may be helpful for immune enhancement. To the best of our knowledge, this is the first study to provide in vivo evidence of immune enhancement by 6MK, which may be helpful for treating different immunity-associated conditions and diseases.

## 2. Materials and Methods

### 2.1. Materials

6MK was obtained from Angene International Ltd., Nanjing, China (CAS Number: 32520-55-1). A stock solution was prepared by dissolving 250 mg 6MK in 1 mL dimethyl sulfoxide. Working concentrations were prepared by serially diluting stock solutions in physiological saline. Antibodies against phosphorylated JNK (p-JNK) (AP0631) and JNK (A4867) were supplied by ABclonal (Boston, MA, USA). Anti-β-actin antibody was purchased from Abcam (Cambridge, UK) (ab6276). Cell Signaling Technology (Boston, MA, USA) provided antibodies targeting p-NF-kB (3033S), NF-κB (8242S), phosphorylated ERK (p-ERK) (4370S), ERK (4695S), phosphorylated p38 (p-P38) (4511S), P38 (8690S), nuclear factor erythroid 2-related factor 2 (Nrf2) (12721S), heme oxygenase-1 (HO-1) (43966S), superoxide dismutase (SOD) (1314S), and catalase (CAT) (14097S) was purchased from Santa Cruz Biotechnology (Dallas, TX, USA).

### 2.2. RAW264.7 Macrophage Cell Culture, Experimental Animals and Study Design

Murine RAW 264.7 macrophages (ATCC, Manassas, VA, USA) were cultured in Dulbecco’s Modified Eagle Medium (DMEM; Corning, NY, USA) supplemented with 10% fetal bovine serum and 1% antibiotic–antimycotic (Gibco, Gaithersburg, MD, USA) at 37 °C in a humidified 5% CO_2_ incubator. Cells were seeded into 96-well plates at a density of 2 × 10^4^ cells/well and allowed to adhere for 24 h. Subsequently, the cells were treated with various concentrations (6.25, 12.5, 25, and 50 µM) of 6MK for an additional 24 h. Afterward, the medium was replaced with fresh medium containing a Cell Counting Kit-8 (CCK-8, Dojindo, Kumamoto, Japan) solution, and the cells were incubated for 2 h at 37 °C. The optical density (OD) was measured at 450 nm using a microplate spectrophotometer (BioTek Inc., Winooski, VT, USA). Cell viability was expressed as a percentage relative to the control group, which was defined as 100% viable.

**Figure 1:**
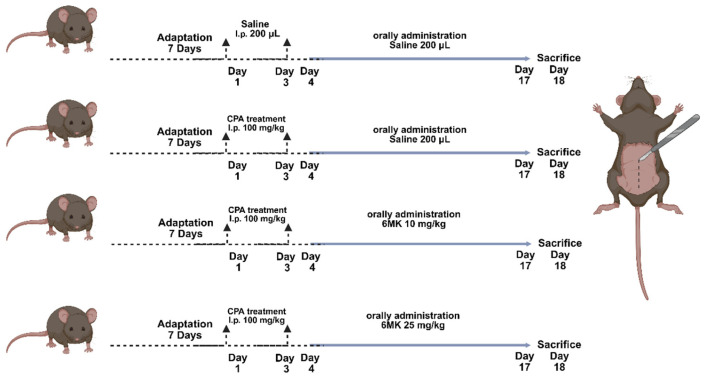
A schematic figure of an animal model experiment. The mice were randomly assigned to four experimental groups (n = 10 per group) as follows: Normal control (NC), cyclophosphamide (CPA) control (CP), CPA + 6MK 10 mg/kg/day (6MK_10), and CPA + 6MK 25 mg/kg/day (6MK_25). 6MK; 6-Methoxy kaempferol.

Five-week-old male BALB/c mice were obtained from Central Lab Animal Inc. (Seoul, Republic of Korea) and acclimated for one week under standard laboratory conditions with ad libitum access to food and water. The animals were housed at a controlled temperature (20–25 °C), humidity (50–55%), and a 12-h light/dark cycle. Following the acclimation period, mice were randomly assigned to four groups (*n* = 10 per group): normal control (NC), CPA control (CP), CPA + 6MK 10 mg/kg/d (6MK_10), and CPA + 6MK 25 mg/kg/d (6MK_25) (Figure 1).

Immunosuppression was induced in all groups except for the NC group via intraperitoneal injection of CPA (100 mg/kg) on days 1 and 3. CPA was dissolved in sterile saline, and mice in the NC group received an equivalent volume of saline. After the final oral administration of 6MK, mice were fasted for 12 h and euthanized by CO_2_ asphyxiation. Blood samples were collected immediately after the euthanasia via cardiac puncture. Thymus and spleen tissues were harvested, weighed, and stored at –80 °C until further analysis. All animal procedures were conducted in accordance with the Guidelines for the Care and Use of Laboratory Animals and were approved by the Institutional Animal Care and Use Committee of Gachon University (Approval No. GU1-2024-IA0015-00).

### 2.3. Measurement of Body Weight and Organ Index

Body weights of the mice were measured five times over 2 weeks. Final body weight was measured 24 h after treatment, before euthanasia. On the day of euthanasia, the thymus and spleen were excised and immediately weighed. The immune organ indices of the thymus and spleen were calculated using the following formula:

Organ index (%)=(Organ weight÷bodyweight)×100

### 2.4. Hematoxylin and Eosin (H&E) Staining

Thymus and spleen tissues were fixed in 10% neutral buffered formalin (Greiner Bio-One CELLSTAR^®^, Frickenhausen, Germany) and processed for paraffin embedding. Tissue sections were stained with H&E to evaluate histological changes. Histopathological analysis was performed using a Provis AX70 light microscope (Olympus, Tokyo, Japan).

### 2.5. Natural Killer (NK) Cell Activity in Blood and Measurement of Hepatic Aspartate Aminotransferase (AST), Alanine Aminotransferase (ALT) and Malondialdehyde (MDA)

NK cell activity was evaluated using whole blood samples. Briefly, 100 μL of blood was collected into heparinized tubes and mixed with 30 μL of NK cell activator (NKMAX; Seongnam-si, Gyeonggi-do, Republic of Korea). The mixture was then incubated at 37 °C for 24 h. After incubation, the samples were centrifuged at 1000× *g* for 15 min to obtain the supernatants. The concentration of interferon-gamma (IFN-γ) in the supernatant was quantified using the Murine NK Cell Activity Kit (NKMAX, Seongnam-si, Gyeonggi-do, Republic of Korea) according to the manufacturer’s instructions. Liver tissue samples (50 mg) were rinsed with phosphate-buffered saline (PBS) (pH 7.4) and homogenized in 1 mL of ice-cold Tris buffer (100 mM, pH 7.8). The homogenates were centrifuged, and the resulting supernatants were collected. AST and ALT activities were then determined using commercial colorimetric assay kits (Asanpharm, Seoul, Republic of Korea) according to the manufacturer’s instructions. For MDA determination, liver tissue (50 mg) was homogenized in 1.15% (*w*/*v*) KCl. The homogenate was mixed with 8.1% sodium dodecyl sulfate, 20% acetic acid (pH 3.5), and 0.8% thiobarbituric acid. The mixture was incubated at 95 °C for 60 min, then cooled to room temperature. Following the addition of n-butanol and distilled water, the samples were centrifuged. The absorbance of the supernatant was measured at 532 nm to quantify MDA.

### 2.6. Splenocyte Proliferation Assay Using Mitogen Stimulation

Splenocytes were immediately isolated from freshly harvested spleens and seeded into 96-well plates at a density of 5 × 10^5^ cells per well in Roswell Park Memorial Institute Medium-1640 medium supplemented with 10% fetal bovine serum (Thermo Fisher Scientific, Waltham, MA, USA). Cells were stimulated with mitogens, concanavalin A (Con A; 5 μg/mL) or lipopolysaccharide (LPS; 1 μg/mL), or left unstimulated as a control, and incubated for 24 h at 37 °C in a humidified 5% CO_2_ atmosphere. Cell viability was assessed by adding 100 μL of Cell Counting Kit-8 (Dojindo Laboratories, Kumamoto, Japan) to each well, following the manufacturer’s instructions. Absorbance was measured at 450 nm using a microplate spectrophotometer.

### 2.7. Measurement of Levels of Nitric Oxide (NO), Cytokines, and Immunoglobulins in Serum and Levels of AST, ALT, and MDA in Liver Tissues

Serum samples were collected via cardiac puncture, centrifuged at 3000 rpm for 10 min at 4 °C, and stored at −80 °C until analysis. NO levels were quantified using the Griess Reagent System (Promega, Madison, WI, USA). Serum cytokine concentrations, including tumor necrosis factor (TNF)-α, interleukin (IL)-1β, IL-6, IL-10, IL-4, and transforming growth factor (TGF)-β, were measured using enzyme-linked immunosorbent assay (ELISA) kits (R&D Systems, Minneapolis, MN, USA). Immunoglobulin levels, including those of IgA, IgM, and IgG, were determined using commercial ELISA kits (Abcam, Cambridge, MA, USA).

### 2.8. Western Blot Analysis

Western blot analysis was performed to investigate the effects of 6MK on splenic tissue protein expression. Total protein was extracted using radioimmunoprecipitation assay buffer (iNtRON Biotechnology, Gyeonggi-do, Republic of Korea) supplemented with protease and phosphatase inhibitors (Sigma-Aldrich, St. Louis, MO, USA; Thermo Scientific, Waltham, MA, USA). Protein concentrations were determined using the BCA Protein Assay Kit (TaKaRa Bio, Kusatsu, Shiga, Japan). Equal amounts of protein (40 μg) were separated by sodium dodecyl sulfate-polyacrylamide gel electrophoresis on 10% or 15% polyacrylamide gels and transferred to polyvinylidene difluoride membranes (Bio-Rad Laboratories, Hercules, CA, USA). The membranes were blocked with 5% skim milk for 2 h at room temperature on a shaker and incubated overnight at 4 °C with primary antibodies. After washing thrice with 1× tris-buffered saline with tween 20 (8 min per wash), the membranes were incubated with horseradish peroxidase-conjugated secondary antibodies for 1 h at room temperature. Protein bands were visualized using an enhanced chemiluminescence detection system with a Quant LAS 500 imaging system (GE Healthcare Bio-Sciences AB, Uppsala, Sweden) following the manufacturer’s protocol. The primary antibodies used included antibodies against the following proteins: p-NF-kB (1:1000), NF-kB (1:1000), p-JNK (1:1000), JNK (1:1000), p-ERK (1:1000), ERK (1:1000), p-p38 (1:1000), p38 (1:1000), SOD (1:2000), catalase (CAT; 1:2000), GPx-1 (1:1000), and β-actin (1:5000).

### 2.9. Molecular Docking Analysis

A molecular docking simulation was conducted to study the binding interaction of 6MK within the active site of the TLR4/MD2 receptors. Euodenine A, a phytochemical that is a known agonist of TLR4/MD2 receptors, was used as positive control [30]. The structures of the ligands (6MK and euodenine A) were extracted from the PubChem database and prepared for docking using the AutoDock Tool kit [31]. The X-ray crystal structure (PDB ID: 3VQ2) of the TLR4/MD2 complex containing LPS was obtained from the PDB database [24]. Water molecules and ligands were removed from the TLR4/MD2 crystal structure and structural minimization was conducted using UCSF Chimera [32]. The entire LPS-binding site spanning the MD2 and TLR4 interacting region was used to define the docking grid in the TLR4/MD2 complex. AutoDock 4.2 was used to conduct the docking simulation, taking ligands as flexible [31]. Different ligand poses were analyzed using the AutoDock toolkit version 1.5.6 and ranked according to the estimated free energy of binding. Interactions between the protein and docked ligand complexes were analyzed by visualization in UCSF Chimera version 1.15 and plotted using LigPlot^+^ version 4.4.0 [33].

### 2.10. Gut Microbiome Sequencing and Analysis

The effect of 6MK on the gut microbiome was studied through metagenome sequencing and analysis of amplicon sequences of the V3–V4 region from 16 S ribosomal sequences. These amplicon sequences were used to construct a library of sequences using the Herculase II Fusion DNA Polymerase Nextera XT Index V2 kit (Santa Clara, CA, USA) for sequencing on an Illumina platform. The paired-end reads obtained from the sequencer were analyzed using the Quantitative Insights into Microbial Ecology (QIIME-2) pipeline [34]. First, paired-end reads imported into the QIIME-2 pipeline were subjected to a quality control step (QCS), including denoising, trimming, and filtering of low-quality reads and chimeras, after visualization of the quality score of the paired-end reads. The divisive amplicon denoising algorithm 2 program was used for QCS [35]. Amplicon sequence variants (ASVs) obtained after QCS were used to study microbial communities. For phylogenetic analysis, multiple sequence alignments were conducted using MAFFT version 7.475 to align ASVs, and a phylogenetic tree was constructed using FastTree version 2.1.10 [34].

Taxonomic annotation of the sequences was performed using the Greengenes 13_8 99% operational taxonomic units (OTU)-based taxonomy classifier, which uses the naïve Bayes algorithm for annotation. Finally, taxonomic annotations were drawn on the bar graphs for each sample. Alpha diversity was calculated based on the richness and evenness of the microbial community in the samples using parameters such as Shannon’s diversity index, observed features, and Faith’s phylogenetic diversity. Community evenness was assessed using Pielou’s evenness measure. Beta diversity of the groups was calculated through qualitative and quantitative measures using different distance methods, including the Jaccard and Bray–Curtis distance measures.

Differentially abundant taxa in the groups were identified using a linear discriminant analysis effect size (LEfSe) program version 1.0 [36]. The species-level taxonomy results obtained from the QIIME2 pipeline were used to identify differentially abundant species in the groups. A strict one-against-all strategy was used in the LEfSe analysis to compare the different groups.

Functional annotation of the gut microbiome community was performed using the PICRUSt2 [37] method which predicts Enzyme Commission numbers (EC numbers), Kyoto Encyclopedia of Genes and Genomes orthologs (KO), and pathway abundance in the samples [37]. Pathway abundance is the main high-level prediction result computed through the structured mapping of EC gene families to pathways. Differentially abundant functions of the microbial communities in the treated and untreated groups were identified through differentially expressed pathways using ALDeX2 version 1.18.0 [38].

### 2.11. Statistical Analysis

Statistical analyses were performed using GraphPad Prism version 10.4.2 (GraphPad Software Inc., La Jolla, CA, USA). One-way analysis of variance was conducted, followed by Tukey’s post hoc multiple comparison test. Data are presented as mean ± standard deviation. Statistical significance was set at *p* < 0.05. Statistical significance is indicated as follows: * *p* < 0.05, ** *p* < 0.01, *** *p* < 0.001, and **** *p* < 0.0001.

## 3. Results

### 3.1. Effects of 6MK on RAW264.7 Macrophage and Body Weight, Immune Organ Index and Hepatic AST, ALT, and MDA Levels in Mice

**Figure 2:**
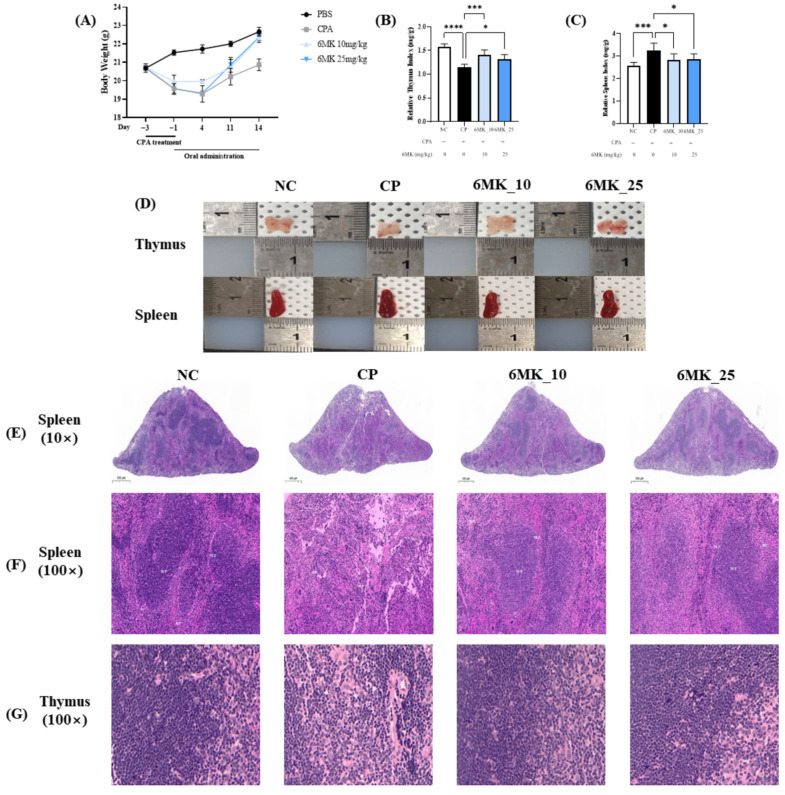
Effects of 6MK on body weight, organ index, and histology of spleen and thymus tissues in cyclophosphamide (CPA)-induced immunosuppressed mice. (**A**) Body weight changes during the experimental period. (**B**) Relative thymus index (mg/g). (**C**) Relative spleen Index (mg/g) (**D**) External appearance of thymus and spleen. (**E**,**F**) spleen tissues (**G**) thymus tissues. Mice were divided into four groups: Normal control (NC), cyclophosphamide control (CP), CPA + 6MK 10 mg/kg/day (6MK_10), and CPA + 6MK 25 mg/kg/day (6MK_25). 6MK; 6-Methoxy kaempferol. Three independent experiments present Data as mean ± standard deviation (SD). * *p* < 0.05, *** *p* < 0.001, and **** *p* < 0.0001 vs. CP group.

Treatment with 6MK at concentrations up to 50 µM had no significant impact on the viability of RAW264.7 macrophages over a 24 h period in the CCK-8 assay, which indicates that these doses may be safe for these cells (Figure S1). Figure 2A shows changes in body weight across all groups during the experimental period. Mice treated with CPA showed significantly reduced body weights than those in the NC group. Notably, the body weight of the mice in the 6MK-treated groups began to recover after 4 d of oral administration, with a more rapid restoration observed relative to the CPA control group. The thymus and spleen were evaluated using organ indices that indirectly indicated systemic immune activity. The results presented as immune organ indices are shown in Figure 2B,C. The thymus and spleen indices in the CPA group were significantly reduced and enhanced, respectively, compared to those in the NC group, whereas 6MK administration significantly restored both the thymus and spleen indices toward the normal state. Levels of liver damage marker enzymes, such as AST and ALT, were not significantly altered in the CPA group compared to the control or treatment groups, suggesting that CPA has no toxic effect on the liver (Figure S2A,B). Similarly, levels of MDA, a marker of oxidative stress, were not significantly altered in the CPA group compared to the control group (Figure S2C).

### 3.2. Effects of 6MK on Histological Alterations in the Spleen and Thymus

Histological examination of the spleen tissues revealed a well-defined boundary between the red and white pulps in the NC group, with densely and orderly arranged lymphoid cells. In contrast, the CP group exhibited structural disruption, including an indistinct boundary between the red and white pulp and an irregular cellular architecture. Notably, the administration of 6MK restored splenic structure, as evidenced by the reestablishment of red–white pulp demarcation and improved cellular organization.

In the thymic tissues, the CP group showed marked atrophy, characterized by a reduction in the cortical area and relative expansion of the medullary region. However, in mice administered with 6MK, the cortical–medullary boundary appeared to be restored, with the tissue architecture resembling that of the NC group (Figure 2G).

### 3.3. Effects of 6MK on NK Cell Activity and Splenocyte Responses in Immunosuppressed Mice

**Figure 3:**
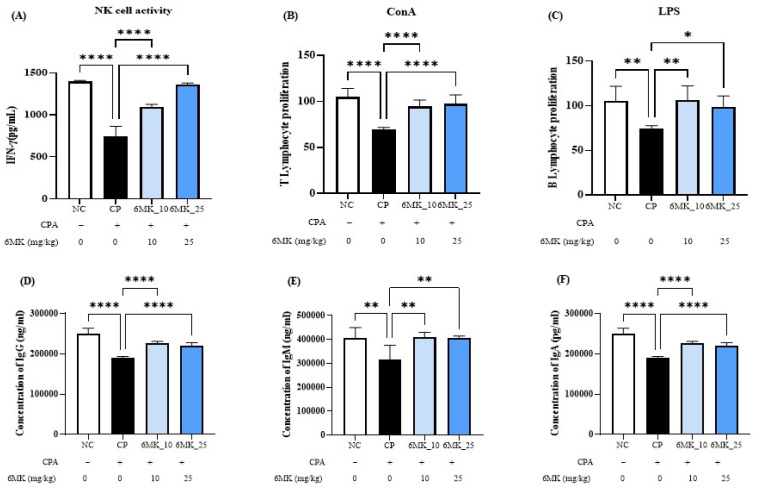
Effect of 6MK on NK cell activity, splenocyte proliferation and serum immunoglobulins (IgG, IgA, and IgM) levels in cyclophosphamide (CPA)-induced immunosuppressed mice. (**A**) IFN-γ levels in peripheral blood were measured to indicate NK cell activity. (**B**,**C**) Splenocyte proliferation was evaluated using the Cell Counting Kit-8 (CCK-8) assay following stimulation with Concanavalin A (Con A) and lipopolysaccharide (LPS), respectively. (**D**) IgG, (**E**) IgM, and (**F**) IgA levels in serum. Three independent experiments present Data as mean ± standard deviation (SD). * *p* < 0.05, ** *p* < 0.01, **** *p* < 0.0001 vs. CP group.

To assess NK cell function, IFN-γ secretion was measured as a key indicator. The CPA group exhibited significantly reduced IFN-γ levels than the NC group, indicating suppressed NK cell activity. However, treatment with 6MK significantly restored IFN-γ secretion (Figure 3A), suggesting a reversal of immunosuppression. In addition, lymphocyte proliferation in the spleen was analyzed to evaluate the cellular immunomodulatory effects of 6MK. Stimulation with Con A and LPS revealed that both T- and B-cell proliferative responses were markedly suppressed in the CP group. Conversely, 6MK treatment significantly increased splenocyte proliferation in response to both mitogens compared to that in the CP group (Figure 3B,C), indicating that 6MK enhances immune responsiveness under immunosuppressive conditions.

### 3.4. Effects of 6MK on the Serum Levels of NO and Cytokines in Immunosuppressed Mice

**Figure 4:**
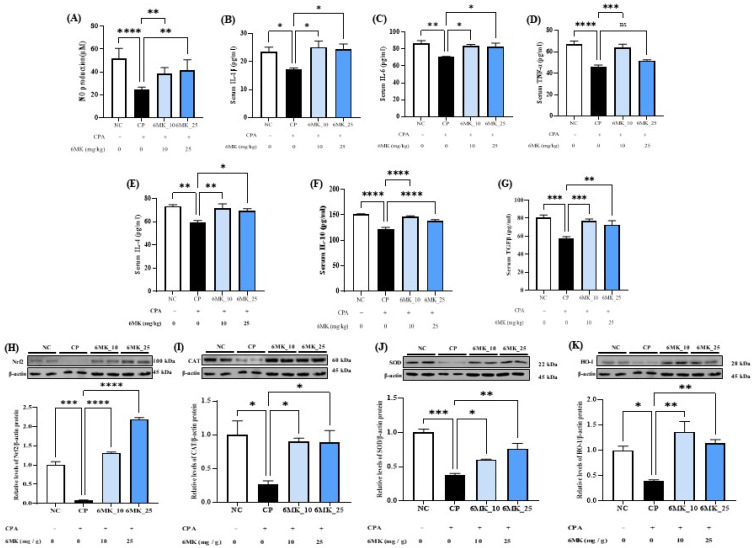
Effects of 6MK on nitric oxide (NO) production, cytokine levels, and expression of antioxidant enzymes in cyclophosphamide (CPA)-induced immunosuppressed mice. (**A**) Serum NO levels. (**B**–**D**) Serum levels of pro-inflammatory cytokines: interleukin-1β (IL-1β), interleukin-6 (IL-6), and tumor necrosis factor-α (TNF-α). (**E**–**G**) Serum levels of anti-inflammatory cytokines: interleukin-4 (IL-4), interleukin-10 (IL-10), and transforming growth factor-β (TGF-β). (**H**) Nuclear factor erythroid 2-related factor 2 (Nrf2), (**I**) catalase (CAT), (**J**) superoxide dismutase (SOD), and (**K**) heme oxygenase-1(HO-1) protein levels in the spleen tissue were measured by Western blot analysis. Mice were divided into four groups: Normal control (NC), cyclophosphamide control (CP), CPA + 6MK 10 mg/kg/day (6MK_10), and CPA + 6MK 25 mg/kg/day (6MK_25). 6MK; 6-Methoxy kaempferol. Three independent experiments present Data as mean ± standard deviation (SD). * *p* < 0.05, ** *p* < 0.01, *** *p* < 0.001, and **** *p* < 0.0001 vs. CP group.

NO, a critical mediator of immune signaling pathways, is predominantly produced by activated macrophages. In the present study, the CPA-treated group showed markedly reduced serum NO levels, indicating suppression of macrophage activation. In contrast, 6MK administration significantly enhanced NO production, indicating its immunostimulatory potential (Figure 4A). To further investigate the macrophage-activating effects of 6MK, serum levels of key cytokines associated with macrophage function, including IL-1β, IL-6, TNF-α, IL-4, IL-10, and TGF-β, were quantified using ELISA. Compared to the CP group, the 6MK group exhibited significantly increased levels of all measured cytokines (except TNF-α levels in high dose treatment), indicating a dose-dependent immunomodulatory effect of 6MK on CPA-induced immunosuppression (Figure 4).

### 3.5. Effects of 6MK on the Serum Levels of IgG, IgA, and IgM in Immunosuppressed Mice

Immunoglobulins are antigen-specific antibody proteins that play essential roles in pathogen neutralization and immunological memory development. This study showed markedly reduced serum immunoglobulin levels in CPA-treated mice, indicating suppression of humoral immunity. However, 6MK administration significantly restored the concentrations of IgA, IgM, and IgG, suggesting its potential to recover CPA-induced impairments in antibody-mediated immune responses (Figure 3D–F).

### 3.6. Effects of 6MK on the Expressions of Antioxidant Enzymes and Transcription Factors in Immunosuppressed Mice

In this study, the antioxidant responses to 6MK were evaluated by assessing the expression levels of Nrf2, CAT, HO-1, and SOD in the spleen. The CP group exhibited significant downregulation in the expression of Nrf2, SOD, CAT, and HO-1 compared to the NC group. Notably, treatment with 6MK restored the expression of these enzymes and transcription factors to levels comparable to those in the NC group, suggesting that 6MK enhances antioxidant defense mechanisms under immunosuppressive conditions (Figure 4H–K).

### 3.7. Effects of 6MK on MAPK Signaling in Immunosuppressed Mice

**Figure 5:**
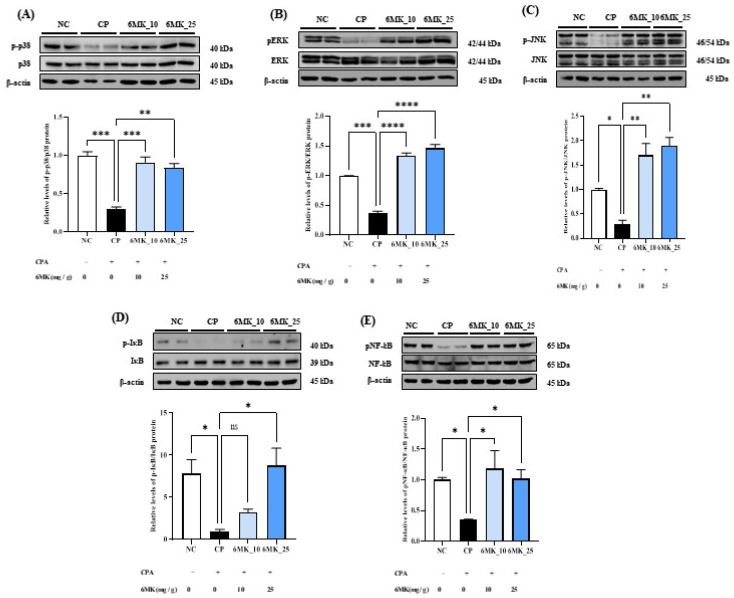
Effect of 6MK on MAPK and NF-κB signaling in cyclophosphamide (CPA)-induced immunosuppressed mice. (**A**) Phosphorylated JNK to total JNK (p-JNK/JNK), (**B**) phosphorylated ERK to total ERK (p-ERK/ERK), (**C**) phosphorylated p38 to total p38 (p-p38/p38), (**D**) Phosphorylated IκBα (p-IκBα) and (**E**) the ratio of phosphorylated NF-κB to total NF-κB (p-NF-κB/NF-κB) protein levels in spleen tissue were measured by Western blot analysis. Mice were divided into four groups: Normal control (NC), cyclophosphamide control (CP), CPA + 6MK 10 mg/kg/day (6MK_10), and CPA + 6MK 25 mg/kg/day (6MK_25). 6MK; 6-Methoxy kaempferol. Three independent experiments present Data as mean ± standard deviation (SD). ns not significant, * *p* < 0.05, ** *p* < 0.01, *** *p* < 0.001, and **** *p* < 0.0001 vs. CP group.

The MAPK signaling pathway is pivotal for mediating diverse cellular responses, including cell proliferation, differentiation, apoptosis, and various biochemical processes. To investigate the molecular mechanisms underlying the effects of 6MK, we examined the expression levels of phosphorylated JNK, ERK, and p38, which are key downstream effectors of the MAPK signaling cascade. As shown in Figure 5, the levels of p-JNK, p-ERK, and p-p38 in the spleen tissues were significantly reduced in CPA-treated mice than those in the NC group. Notably, treatment with 6MK markedly increased the phosphorylation levels of JNK, ERK, and p38, indicating that 6MK activates the MAPK signaling pathway in immunosuppressed mice.

### 3.8. Effect of 6MK on NF-κB Signaling in Immunosuppressed Mice

NF-κB plays a central role in orchestrating both innate and adaptive immune responses. To investigate the regulatory effect of 6MK on the NF-κB signaling pathway, we assessed the phosphorylation levels of NF-κB and inhibitor of NF-κB (IκB) in spleen tissues. In the CPA-induced immunosuppressed group, the phosphorylation levels of both NF-κB and IκB were significantly decreased compared to those in the NC group. However, treatment with 6MK significantly restored the phosphorylation of these proteins, suggesting activation of the NF-κB pathway and potential reversal of immunosuppression (Figure 5D,E).

### 3.9. 6MK Docking and Interaction with TLR4/MD2 Receptors

**Figure 6:**
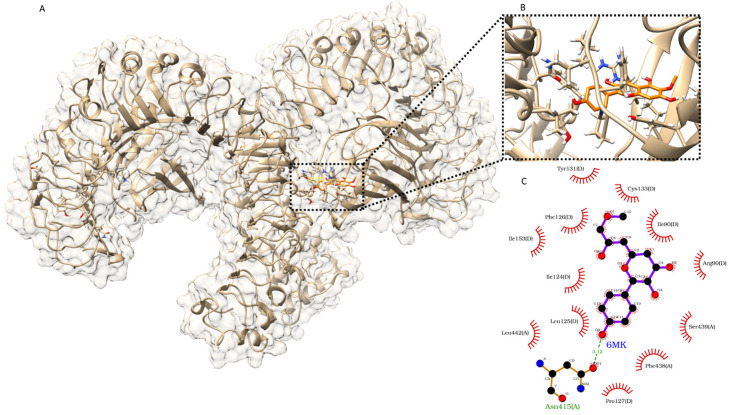
Binding and interaction of 6MK docked in the TLR4/MD2 complex. (**A**) Full-length structure of the TLR4/MD2 complex with docked 6MK. (**B**) Close-up view of docked 6MK within the TLR4/MD2 complex binding site. (**C**) Two-dimensional depiction of the interaction of 6MK with the TLR4/MD2 complex, as identified by LigPlot^+^.

In the docking analysis, the estimated free energy of binding of 6MK was found to be −7.20 kcal/mol, which was slightly more than the positive control (−8.22 kcal/mol) used in the study. During visualization of the docking pose, the binding location of 6MK was found to be at the interacting region of TLR4 and MD2. LPS binds to the TLR4/MD2 complex at the same binding site to stimulate an immune response. Interaction analysis revealed that 6MK interacted with both TLR4 and MD2 receptors. A total of 12 hydrophobic interactions and one hydrogen bond were found between the 6MK and target TLR4/MD2 receptor complex (Figure 6B,C). The hydrogen bond interaction was observed between 6MK and asparagine 415 in TLR4 cells (Figure 6C). The hydrogen bond interaction is a directional interaction that may contribute to ligand specificity. Additionally, 6MK showed three and nine hydrophobic interactions with MD2 and TLR4, respectively, which may have contributed to its high affinity toward the TLR4/MD2 receptor complex. Finally, the agonistic potential of 6MK in the docking analysis was supported by its comparable docking score to the known agonist euodenine A and the large number of shared interactions at the LPS-binding site residue (Figure S3).

### 3.10. Gut Microbiome Diversity Analysis

**Figure 7:**
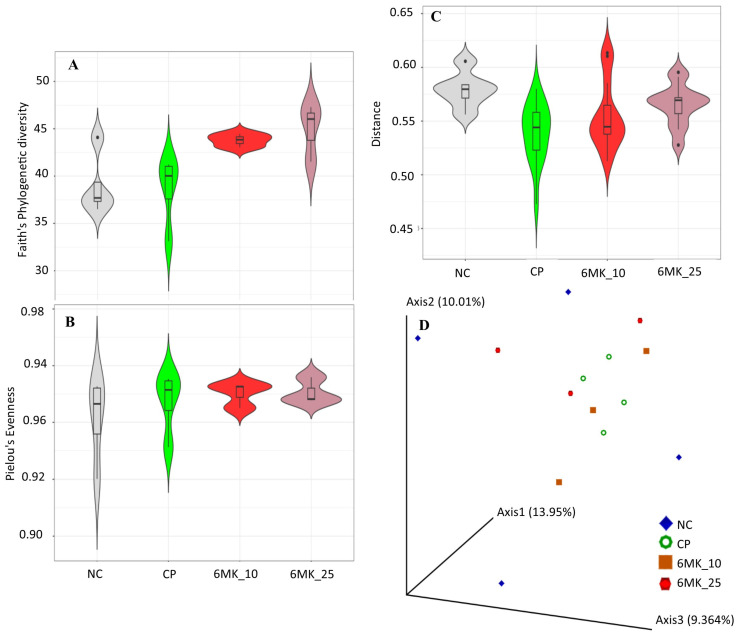
Gut microbiome analysis results. (**A**) Alpha diversity: Faith’s Phylogenetic Diversity. (**B**) Alpha diversity: Pielou’s Evenness Index. (**C**) Beta diversity plot, control group used as the reference for distance calculation. (**D**) Three-dimensional plot of beta diversity based on the unweighted UniFrac distance. Normal control (NC), cyclophosphamide control (CP), CPA + 6MK 10 mg/kg/day (6MK_10), and CPA + 6MK 25 mg/kg/day (6MK_25). 6MK; 6-Methoxy kaempferol.

After QCS, 566,413 ASVs/feature reads/OTUs were used for the analysis, comprising 17,116 unique features/ASVs identified in all groups. An increase in faith phylogenetic diversity (alpha diversity) was observed in the treatment groups, which was significant between the CPA and treatment groups (Figure 7A). No significant changes in alpha diversity evenness were observed between groups (Figure 7B). Similarly, changes in the beta diversity were not significant according to permutational multivariate analysis of variance (Figure 7C). The 3D graph of beta diversity based on the unweighted UniFrac distance showed that the samples of the CPA groups were concentrated in the middle part of the graph, and the samples of the treatment and control groups were relatively dispersed in the graph (Figure 7D).

### 3.11. Gut Microbiome Taxonomy Analysis

The taxonomic annotation of the samples revealed that the gut microbiome of most samples belonged to the phylum Firmicutes, followed by Bacteroidetes (Figure S4). Clostridia was the most abundant class in all samples, followed by Bacteroidia. Clostridiales was the most abundant order in all samples, followed by Bacteroidales.

### 3.12. Differentially Abundant Microbial Community

**Figure 8:**
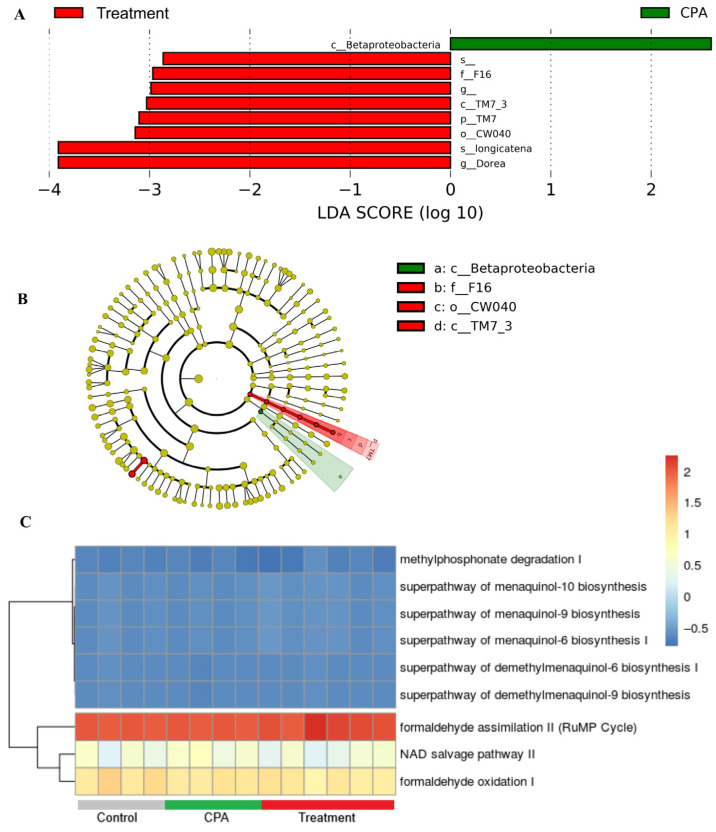
Differentially abundant taxa in gut microbiome. (**A**) Bar plot depicting abundant taxa (**B**) Cladogram and (**C**) Heatmap showing the abundance of selected pathways, which were differentially abundant in the 6MK treatment.

Species-level differential abundance analysis based on the Linear Discriminant Analysis method revealed that *Dorea longicatena*, a Gram-positive bacterium, was more abundant in the treatment group (Figure 8A). Furthermore, phylum TM7, also known as Saccharibacteria, was abundant in the treatment groups (Figure 8). The immunomodulatory function of *D. longicatena* has been suggested because as a member of the phylum Firmicutes, it produces different SCFAs, specifically butyrate, which have immunomodulatory functions [27,29]. However, the direct role of *D. longicatena* in immune enhancement has not been studied. The abundance of *D. longicatena* in the gut microbiome has been studied under critical diseases and conditions. Its abundance is negatively associated with metabolic dysfunction-associated fatty liver disease (MAFLD) [39,40]. It also negatively correlates with insulin resistance markers in postmenopausal women [41]. Another study investigating acute graft-versus-host disease found that *D. longicatena* was the most consistently associated candidate species with a positive response to steroid treatment [42]. A previous study suggested an association between *D. longicatena* and obesity [43]. Recent research has demonstrated that the presence of *D. longicatena* is associated with an increase in appendicular lean mass. Therefore, the study suggested that the therapeutic potential of *D. longicatena* and other abundant species against sarcopenia and osteoporosis should be evaluated in future studies [44]. Considering the positive impact of *D. longicatena* on different critical diseases and conditions, studying its role in immune enhancement in the future would be interesting.

### 3.13. Predictive Functional Profiling of Gut Microbiome and Comparison of Treated and Untreated Groups

The functional profile of the gut microbiome was predicted in terms of EC number, KO, and pathways through ASVs. Pathway abundances were the main high-level prediction outputs calculated through structured mapping of EC gene families to pathways. Eight pathways were found to be differentially abundant according to (*p* < 0.05) Welch’s rank test in the comparison of 6MK treated and untreated groups. Of these eight pathways, two pathways (formaldehyde oxidation I and formaldehyde assimilation II [RuMP cycle]) were found to be differentially abundant according to both (*p* < 0.05) Welch’s and Wilcoxon rank tests (Figure 8C and Table S1). Notably, according to Benjamini–Hochberg’s corrected *p*-value, no pathways were found to be differentially abundant. Similarly, 47 KO and 20 EC numbers were found to be differentially abundant according to both (*p* < 0.05) Welch’s and Wilcoxon rank tests (Tables S2 and S3 and Figures S5 and S6).

## 4. Discussion

The recent increase in chronic illnesses associated with immunosenescence, adoption of Western-style diets, and increased vulnerability to infections, particularly highlighted by the Coronavirus disease 2019 pandemic, underscores the critical importance of sustaining and modulating immune function as a strategy for effective disease prevention [45]. The immunomodulatory mechanisms of flavonoids present in plant-based foods remain an active area of research. The immunomodulatory activity of 6MK, which is structurally similar to various flavonoids with multiple pharmacological activities, was studied in a CPA-induced immunosuppressed mouse model [13,14]. CPA targets multiple immune-related cells and organs including the thymus and spleen. In the present study, CPA administration led to a reduction in the thymus size and histopathological distortions, including a blurred distinction between the cortex and medulla, as previously reported (Figure 2E,F). The thymus plays a critical role in the immune system, serving as the primary site for the differentiation of immature T cells into functional mature T lymphocytes. It is also essential for establishing central immune tolerance through the education of T cells to distinguish self from non-self, thereby preventing autoimmunity. As 6MK administration effectively alleviated CPA-induced tissue damage in the thymus (Figure 2D,G), it may be helpful against immune suppression and the risk of autoimmune diseases [46].

The spleen, another critical immune organ, plays a central role in filtering foreign substances and metabolic waste from the bloodstream, and storing and activating immune cells to coordinate systemic immune responses. CPA-induced immunosuppression resulted in splenomegaly due to histological distortion and pathological tissue expansion (Figure 2C–F) [47]. H&E staining of the splenic tissue revealed that the boundary between the white and red pulps was significantly less defined in the CP group than in the NC group (Figure 2E,F). This disorganization reflects the structural impairment of lymphoid tissue and the loss of organized immune cell architecture, both of which are hallmarks of CPA-induced immunosuppression [47]. The significant improvement in the histological structure by 6MK suggests that it may contribute to the recovery of immune organ function under immunosuppressed conditions.

CPA administration did not induce significant hepatic damage, as evidenced by the lack of substantial alterations in liver marker enzymes (AST and ALT) relative to the control and treatment groups (Figure 2A,B). This was further supported by the absence of a significant impact on MDA levels, a marker of oxidative stress (Figure 2C). Consequently, these results suggest that the immunomodulatory effects of 6MK are likely independent of hepatotoxicity reversal. NK cells are innate granular lymphocytes that are vital for early host defense and rapid elimination of infected and tumor cells. They achieve this via cytotoxicity and by secreting IFN-γ, a key cytokine that promotes macrophage activation and Type 1 T helper (Th1) cell polarization; therefore, IFN-γ is used as a functional biomarker for NK cell activity. Our results showed that 6MK effectively counteracted CPA-induced immunosuppression by significantly restoring the diminished NK cell activity in the CP group (Figure 3A), consistent with dietary flavonoid-mediated immune stimulation [48]. This underscores the immunomodulatory potential of 6MK, similar to those of dietary flavonoids against malignant and viral diseases [48].

CPA, which suppresses cellular immunity by inhibiting B- and T-lymphocyte proliferation, significantly reduced splenic cell activation (stimulated by Con A/LPS) in the CP group (Figure 3). Conspicuously, 6MK markedly restored B- and T-lymphocyte proliferation, highlighting its potential to mitigate immunosuppression by modulating immune lymphocyte activity (Figure 3).

Macrophages are key immune mediators with two main functional phenotypes, pro-inflammatory M1 (which helps in pathogen clearance) and anti-inflammatory M2 (which helps in immune regulation and tissue repair), which are associated with pro- and anti-inflammatory cytokines, respectively [49]. Th1 and Th2 cells also secrete pro- and anti-inflammatory cytokines that regulate M1/M2 differentiation, thereby ensuring immune homeostasis [49]. In the present study, levels of pro-inflammatory (TNF-α and IL-1β), anti-inflammatory (IL-4 and IL-10), and both pro- and anti-inflammatory (TGF-β and IL-6) cytokines were significantly suppressed in the CP group, suggesting immunosuppression and loss of immune homeostasis (Figure 4). Notably, the levels of these cytokines were significantly restored by 6MK treatment, indicating a reversal of the CPA-induced suppression (Figure 4). These findings suggest that 6MK enhances or regulates immune function under immunosuppressive conditions by modulating cytokine-mediated signaling pathways.

NO is a critical signaling molecule in the immune system and is upregulated in immune cells in response to immunological stimuli. NO plays a vital role in the elimination of pathogens and regulation of immune cell function, thereby contributing to the maintenance of immune homeostasis [50]. In the present study, NO levels were significantly reduced in the CP group compared to those in the NC group, indicating suppressed immune activity (Figure 4A). However, the significant increase in NO production by 6MK suggests that 6MK may restore immune cell function and augment antimicrobial immune responses under immunosuppressive conditions.

Immunoglobulins produced by B-lymphocytes play a crucial role in the immune system by specifically recognizing antigens and mediating various immune responses. Antibodies are used as biomarkers to study immunity against different diseases and as therapeutics against cancer and infectious diseases [51,52]. Herbal immune stimulants and their phytochemicals enhance immunity by activating B cells, thereby increasing antibody production [53]. In our study, compared with the NC group, the CP group exhibited significantly reduced IgA, IgG, and IgM immunoglobulin levels, reflecting suppressed B cell function. Notably, the 6MK treatment group showed a significant restoration of immunoglobulin levels (Figure 3). These findings suggest that 6MK enhances B cell activation and promotes antibody production, restoring or strengthening humoral immune responses under immunosuppressive conditions.

CPA generates ROS that contribute to oxidative stress and immunosuppression by impairing the activity of endogenous antioxidant enzymes. In the present study, CPA suppressed the protein expression levels of key antioxidant enzymes (SOD, CAT, and HO-1) and a transcription factor (Nrf2) in the spleen compared with those in the NC group, indicating oxidative stress. However, 6MK administration significantly restored the expression of these antioxidant enzymes and transcription factors, reflecting its potent antioxidant capacity. The non-enzymatic antioxidant activity of 6MK through DPPH and ABTS assays has been reported in the literature [10]. Therefore, these results strongly suggest the antioxidant properties of 6MK mediated through enzymatic antioxidant activity, which may contribute to the immune-enhancing activity observed in our study. Knowledge of the molecular mechanisms of a therapeutic agent is essential for understanding its functions, which are crucial for achieving optimal activity and facilitating its development. The MAPK and NF-κB signaling pathways are associated with different key cellular processes, including immunity; therefore, the key proteins of these pathways were studied to explore the underlying molecular mechanism of 6MK-associated immune enhancement.

NF-κB is a key transcription factor that is a central regulator of inflammation and immune responses. It integrates a wide array of extracellular and intracellular stimuli to regulate the expression of numerous cytokines and immunoregulatory genes. Under resting conditions, NF-κB resides in the cytoplasm as an inactive complex bound to its inhibitor, IκB [54]. Upon stimulation by immune mediators, the IκB kinase (IKK) complex phosphorylates IκBα, leading to its degradation and the subsequent release of NF-κB. The freed NF-κB then translocates to the nucleus, where it initiates transcription of its target genes [54]. Activated NF-κB has been shown to play a pivotal role in the activation, differentiation, and proliferation of both T and B lymphocytes, thereby contributing to the coordination of innate and adaptive immune responses. It also regulates the expression of both pro- and anti-inflammatory cytokines and is crucial for the survival, proliferation, and differentiation of immune cells, thereby playing a central role in maintaining the immune system balance [54]. In this study, NF-κB activity (phosphorylation of NF-κB and IκBα) was significantly reduced in the CP group compared to the NC group (Figure 5), along with the suppression of key cytokines, B-cell proliferation activity, and suppression of antibody levels (Figure 3, Figure 4 and Figure 5). This suppression likely reflects the inhibition of NF-κB signaling in the immunocompromised state induced by CPA, which impairs its function as a key regulatory node in immune responses. In contrast, the 6MK-treated group showed a statistically significant restoration of NF-κB activity (phosphorylation of NF-κB and IκBα) compared to the CP group (Figure 5). In future studies, NF-κB nuclear translocation may be assessed via immunofluorescence to further validate NF-κB activation. In the MAPK pathway, JNK, p38, and ERK are the three major subfamilies of the MAPK family. JNK and p38 are primarily activated in response to cellular stressors such as oxidative stress, genotoxic damage, osmotic imbalance, and inflammatory cytokines such as TNF-α and IL-1β [55]. In contrast, ERK is typically activated by growth factors and plays a pivotal role in the regulation of oxidative stress responses and immune function [56].

In this study, the activity of the MAPK signaling pathway was significantly reduced in the CP group compared to that in the NC group, indicating the suppression of this critical immune and stress-response pathway. The observed alterations in antioxidant enzyme expression and inflammatory cytokine profiles supported the hypothesis that CPA induces oxidative stress, which may suppress MAPK pathway activity. Restoration of MAPK signaling in the 6MK group implies that 6MK mitigates CPA-induced oxidative damage and may regulate immune responses by modulating MAPK pathway components. The observed activation of immune-related MAPK and NF-κB signaling pathways by 6MK led us to investigate its interaction with the TLR4/MD2 receptor complex, which is an upstream activator of these pathways. Activation of the TLR4/MD2 receptor complex by agonist binding (such as LPS) enhances the immune response via the stimulation of both the MAPK and NF-κB signaling pathways.

**Figure 9:**
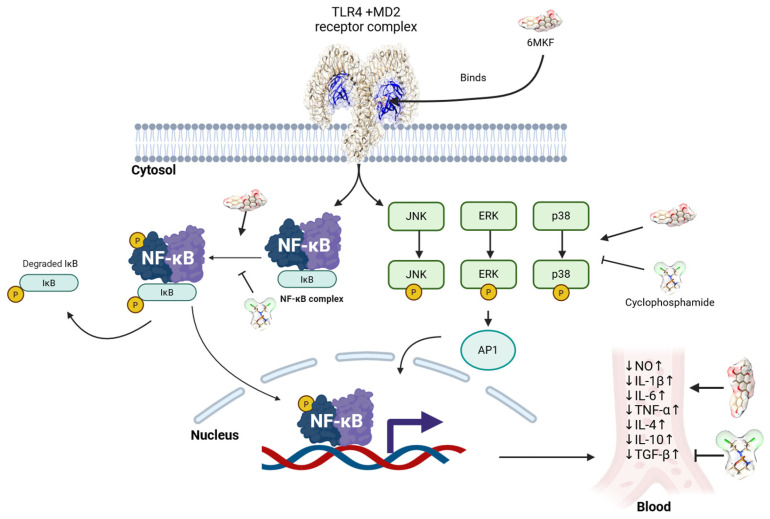
Possible activation of TLR4/MD2 receptor complex and stimulation of NF-κB and MAPK pathways for immune enhancement.

TLR-mediated NF-κB activation is a phylogenetically conserved paradigm of innate immunity [57]. LPS is an important component of the Gram-negative bacterial cell wall that can trigger immune responses by binding to and activating TLR4/MD2 receptors, which have diverse immunomodulatory effects. Because euodenine A activates the TLR4/MD2 receptor to enhance immune responses, we compared its binding to that of 6MK. The similar estimated free energy of binding found in our docking study suggests that 6MK also has agonist potential for the TLR4/MD2 receptors. Interaction analysis of the 6MK docked complex and co-crystallized agonists (such as LPS) present in the crystal structure of the TLR4/MD2 receptor complex revealed that the binding site and important interacting residues (such as Ile80, Arg90, Ile124, Phe126, and Tyr131) were similar to those of LPS. This finding further emphasizes the agonistic potential of 6MK (Figure S3), though it is not sufficient to definitively establish this effect. A strong interaction pattern, which included 12 hydrophobic and hydrogen bond interactions, suggested high affinity and specificity of 6MK for TLR4/MD2 receptors (Figure 6C). However, further analysis, including crystallographic experiments, is required to establish the strong interaction between 6MK and the activation of TLR4/MD2 receptors. Activation of TLR4/MD2 receptors can initiate the signaling cascade to stimulate NF-κB pathway signaling. The possible activation of TLR4/MD2 receptors complex via 6MK binding and observed stimulation of the MAPK and NF-κB pathway signaling responsible for immune enhancement are depicted in Figure 9. Importantly, TLR4 expression exhibits sexual dimorphism in specific immune cells (such as neutrophils and peritoneal macrophages) and organs and is modulated by sex hormones [58,59]. This divergence is known to drive distinct pathophysiological mechanisms in immune-associated diseases between males and females [59,60]. Given that 6MK may exert its effects via the TLR4 pathway, it is imperative to include female mice in future studies, particularly those investigating immune-related pathologies, to accurately capture these sex-specific responses. However, this study has limitations, particularly in the analysis of immune cell subsets. This has not been sufficiently studied for immune cell subpopulations such as specific T- and B-cell subsets. Hematological parameters, such as platelet count, were not considered in the current study. However, these parameters are important for immune function, and they should be analyzed in future studies. Further studies are required to fully elucidate the cellular and molecular mechanisms underlying the immunomodulatory effect of 6MK.

The impact of the gut microbiome on the immune system is well known; however, the association between immunity and the gut microbiome is complex. Gut microbiome analysis revealed no significant differences in beta diversity between groups. However, a significant increase was observed in alpha diversity with 6MK treatment compared to the CPA group, which may be a contributing factor to the immune enhancement observed in the study. An increase in alpha diversity in the absence of significant beta diversity shifts suggests that microbial variations were confined to low-abundance taxa, leaving the dominant community structure largely unaffected. This indicates that the core microbial structure remained stable, suggesting that 6MK does not induce significant dysbiosis within the gut microbiome [61]. Furthermore, differential abundance analysis confirmed that no major bacterial species in the gut microbiome were significantly altered (Figure 8A). Different therapeutic candidates that ameliorate immune suppression in mouse models have shown an increase in alpha diversity without a significant effect on beta diversity [62,63].

Differential abundance analysis revealed the presence of *D. longicatena* in the treatment groups. The direct effects of *D. longicatena* on immunity have not yet been studied. However, *D. longicatena* produces butyrate, which enhances innate immunity by promoting macrophage differentiation, and adaptive immunity by stimulating regulatory T cell (Treg) differentiation and antitumor CD8+ T cell responses [28,29]. Immune enhancement can contribute positively to various diseases and conditions. The positive effects of *D. longicatena* abundance in the gut have been observed in critical diseases (sarcopenia, osteoporosis, diabetes, MAFLD, and acute graft-versus-host disease), emphasizing its possible role and the need to evaluate its effects on immune enhancement [39,40,41,42].

Additionally, the prediction of the functional potential of the microbial community using differential function analysis revealed that the formaldehyde oxidation I and formaldehyde assimilation II (RuMP cycle) pathways were upregulated in the treatment group (Figure 8C,G). These pathways consume formaldehyde, which induces regulatory T cell-mediated immunosuppression [64]. Thus, the upregulation of these pathways reduces formaldehyde levels and may subsequently reduce the immune suppression induced by formaldehyde [64].

Finally, 6MK partially targets NF-κB and MAPK pathways for its enhancement activity. The effect of *D. longicatena* via butyrate production during immune enhancement and the role of formaldehyde-consuming pathways in 6MK-mediated immune enhancement activity should be verified and explored in future studies. Importantly, a 6MK-only group and a pre-treatment arm were not evaluated in the current study, which may be considered a limitation. Given that 6MK is a natural compound with significant immunomodulatory potential, investigating its prophylactic effects could strengthen its candidacy as a supplement. Future studies could incorporate a pre-treatment arm and a 6MK-only group to evaluate its protective role and compare efficacy across both prophylactic and therapeutic dosing schedules.

Further understanding of the underlying molecular mechanisms is crucial for developing 6MK as a therapeutic agent or a supplement for immune enhancement. Further studies evaluating the pharmacological activities of 6MK against immune- and inflammation-related diseases are strongly recommended. Further studies may utilize human peripheral blood mononuclear cells (PBMCs) to determine if the immunomodulatory effects of 6MK are reproducible in a human system, thereby validating the proposed mechanism of action. As 6MK is currently in the early preclinical stage and lacks a comprehensive toxicity and safety profile, systematic pharmacokinetic (PK) and pharmacodynamic (PD) evaluations in animal models are essential to establish its translational potential. Beyond defining the mechanism of action, characterizing the time-course of its effects through PK/PD modeling is essential to establish the dose–response relationship and safety profile required for future clinical trials.

## 5. Conclusions

This study demonstrated that 6MK, a flavonoid derived from sweet cherries, significantly improved immune function in a CPA-induced immunosuppressed mouse model. Administration of 6MK restored the weight of the immune organs, enhanced NK cell cytotoxicity, stimulated lymphocyte proliferation, and increased the levels of cytokines, nitric oxide, immunoglobulins, and antioxidant enzymes. Furthermore, 6MK reactivated suppressed immune-related signaling pathways, including NF-κB and MAPK. The activation of upstream TLR4/MD2 receptors remains speculative and needs to be validated in future studies. The impact on the gut microbiome, including an increase in alpha diversity, abundance of *D. longicatena*, and upregulation of formaldehyde-consuming pathways observed in the treatment group, also supports immune enhancement. These findings suggest that 6MK exerts potent immunomodulatory effects by mitigating oxidative stress and regulating immune signaling cascades. Therefore, 6MK has strong potential as a natural immune-enhancing agent to prevent or alleviate immunosuppression.

## Data Availability

The high-throughput sequencing datasets generated and used in this study have been submitted to a publicly accessible NCBI repository (BioProject: PRJNA1369649).

## Supplementary Materials

The following supporting information can be downloaded at: https://www.mdpi.com/article/10.3390/antiox15030334/s1, Figure S1: Effects of 6MK on RAW 264.7 cells viability. The cells were treated with various concentrations (6.25, 12.5, 25, and 50 µM) of 6MK, and cell viability was assessed through CCK-8 assay; Figure S2: Effects of 6MK on hepatic (AST), (ALT), and malondialdehyde (MDA) levels in a cyclophosphamide (CPA)-induced immunosuppressed mouse model; Figure S3: Common interacting residues of the TLR4/MD2 complex with LPS and 6MKF (shown in dotted circles); Figure S4: Bar plot showing the taxonomic distribution across all samples; Figure S5: Heatmap showing the abundance of enzymes (EC numbers), which were differentially abundant in the 6MKF treatment; Figure S6: Heatmap showing the abundance of KO, which were differentially abundant in the 6MKF treatment; Table S1: Results of comparative pathway abundance analysis through ALDEx2; Table S2: Results of comparative abundance of EC numbers through ALDEx2; Table S3: Results comparative KO abundace analysis through ALDEx2.

## Abbreviations

The following abbreviations are used in this manuscript:

6MK	6-methoxykaempferol
ABTS	2,2′-azinobis(3-ethylbenzothiazoline-6-sulfonic acid)
ASVs	Amplicon sequence variants
CAT	catalase
CCK-8	Cell Counting Kit-8
ConA	Concanavalin A
CP	CPA control
CPA	Cyclophosphamide
DPPH	2,2-diphenyl-1-picrylhydrazyl
EC numbers	Enzyme Commission numbers
ELISA	Enzyme-linked immunosorbent assay
H&E	Hematoxylin and eosin
HO-1	Heme oxygenase-1
IFN-γ	Interferon-gamma
IKK	IκB kinase
IL	Interleukin
IκB	Inhibitor of NF-κB
JNK	c-Jun N-terminal kinase
KO	Kyoto Encyclopedia of Genes and Genomes orthologs
LEfSe	Linear discriminant analysis effect size
LPS	Lipopolysaccharide
MAFLD	Metabolic dysfunction-associated fatty liver disease
MAPK	Mitogen-activated protein kinase
NC	Normal control
NF-κB	Nuclear factor-κB
NK	Natural killer
NO	Nitric oxide
Nrf2	Nuclear factor erythroid 2-related factor 2
OTU	Operational taxonomic units
p-ERK	Phosphorylated ERK
p-JNK	Phosphorylated JNK
p-P38	Phosphorylated p38
QCS	Quality control step
QIIME	Quantitative Insights into Microbial Ecology
ROS	Reactive oxygen species
SOD	superoxide dismutase
TGF-β	Transforming growth factor
Th1	Type 1 T helper
TLR4/MD2	Toll-like receptor-4/myeloid differentiation factor-2 receptor complex
TNF-α	Tumor necrosis factor
Treg	Regulatory T cell
