# Supplementary material for: Immune-Enhancement Effects of 6-Methoxykaempferol on Cyclophosphamide-Induced Immunosuppression via Improving Antioxidant Enzyme Expression, NF-κB and MAPK Signaling, and Modulating Gut Microbiome
Source: Antioxidants (Basel). 2026 Mar 6;15(3):334. doi: 10.3390/antiox15030334 (PMC13024305; doi:10.3390/antiox15030334)
Supplement: Supplementary file 1 [file antioxidants-15-00334-s001.zip › SupplimentaryFiguresS1-S4.pdf]

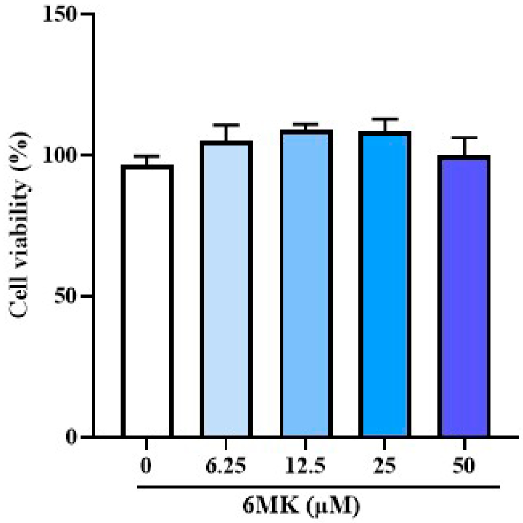

Figure S1. Effects of 6MK on RAW264.7 cells. The cells were treated with various concentrations (6.25, 12.5, 25, and 50 μM) of 6MK, and cell viability was assessed through the CCK-8 assay.

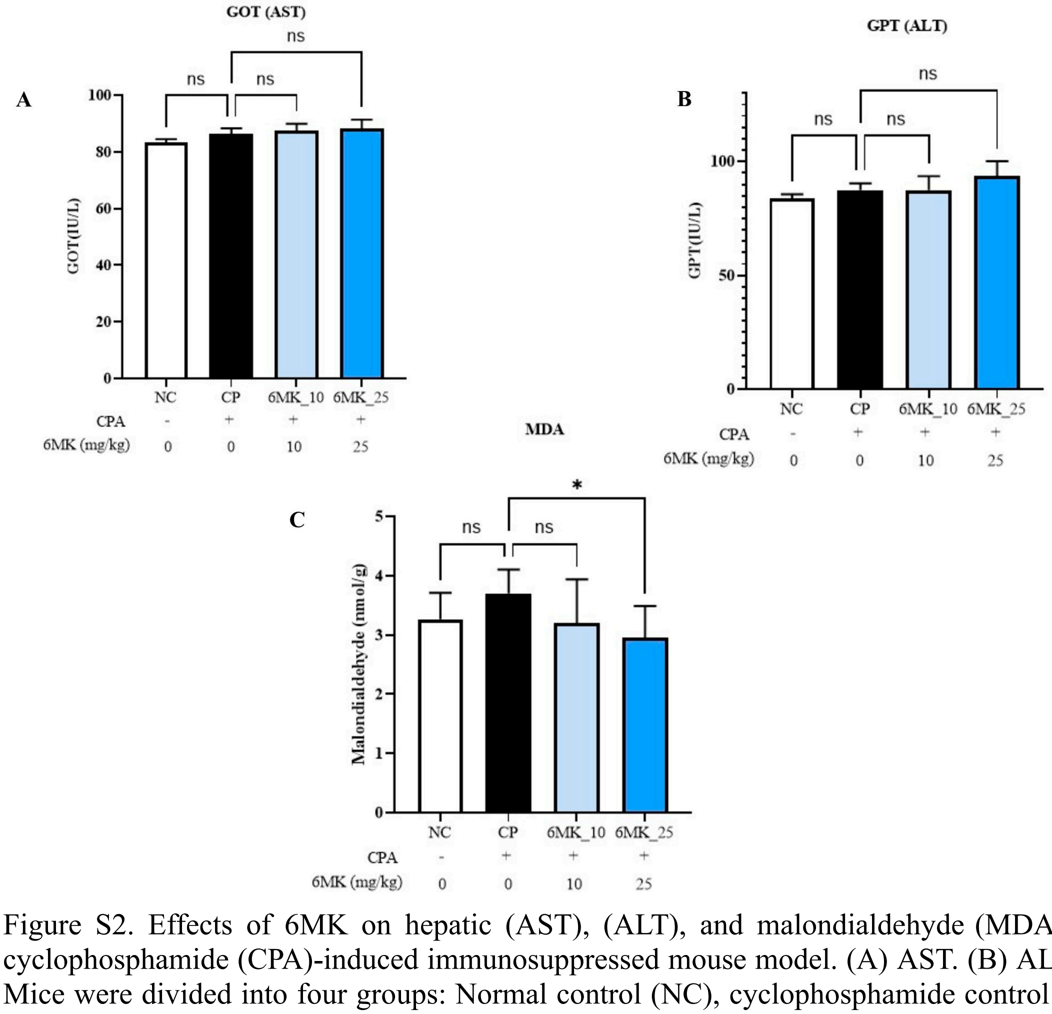

Figure S2. Effects of 6MK on hepatic (AST), (ALT), and malondialdehyde (MDA) levels in a cyclophosphamide (CPA)-induced immunosuppressed mouse model. (A) AST. (B) ALT. (C) MDA. Mice were divided into four groups: Normal control (NC), cyclophosphamide control (CP), CPA + 6MK 10 mg/kg/day (6MK_10), and CPA + 6MK 25 mg/kg/day (6MK_25). 6MK; 6-Methoxy kaempferol. Three independent experiments present Data as mean ± standard deviation (SD). * $p < 0.05$, *** $p < 0.001$, and **** $p < 0.0001$ vs. CP group.

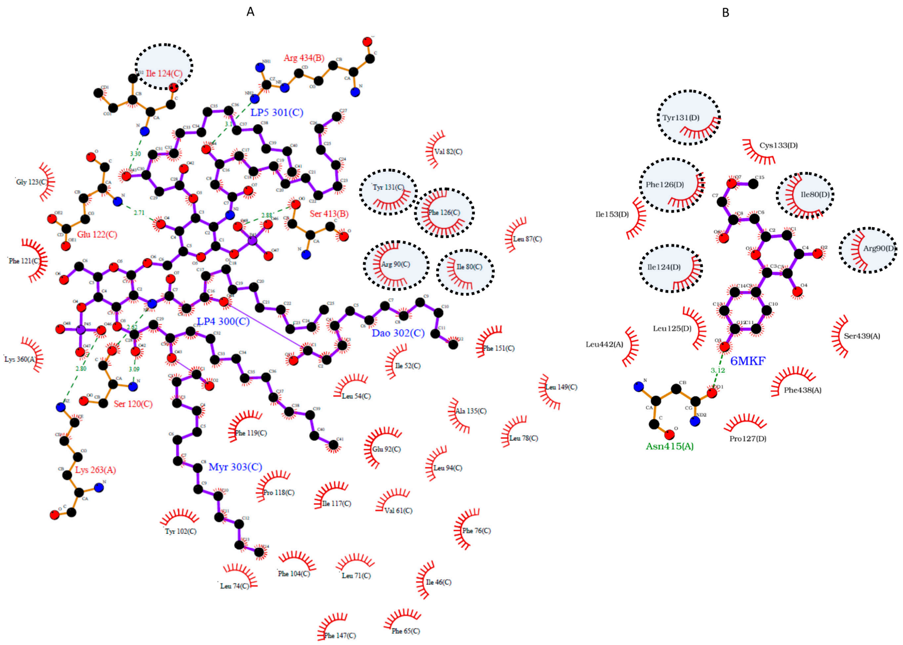

Figure S3. Common interacting residues of the TLR4/MD2 complex with LPS and 6MKF (shown in dotted circles). A: Interacting residues of the TLR4/MD2 complex with LPS derived from the crystal structure of the TLR4/MD2 complex with LPD (PDB ID: 3QV2). B: Interacting residues of the TLR4/MD2 complex with 6MKF derived from the docking complex through LigPlot+.

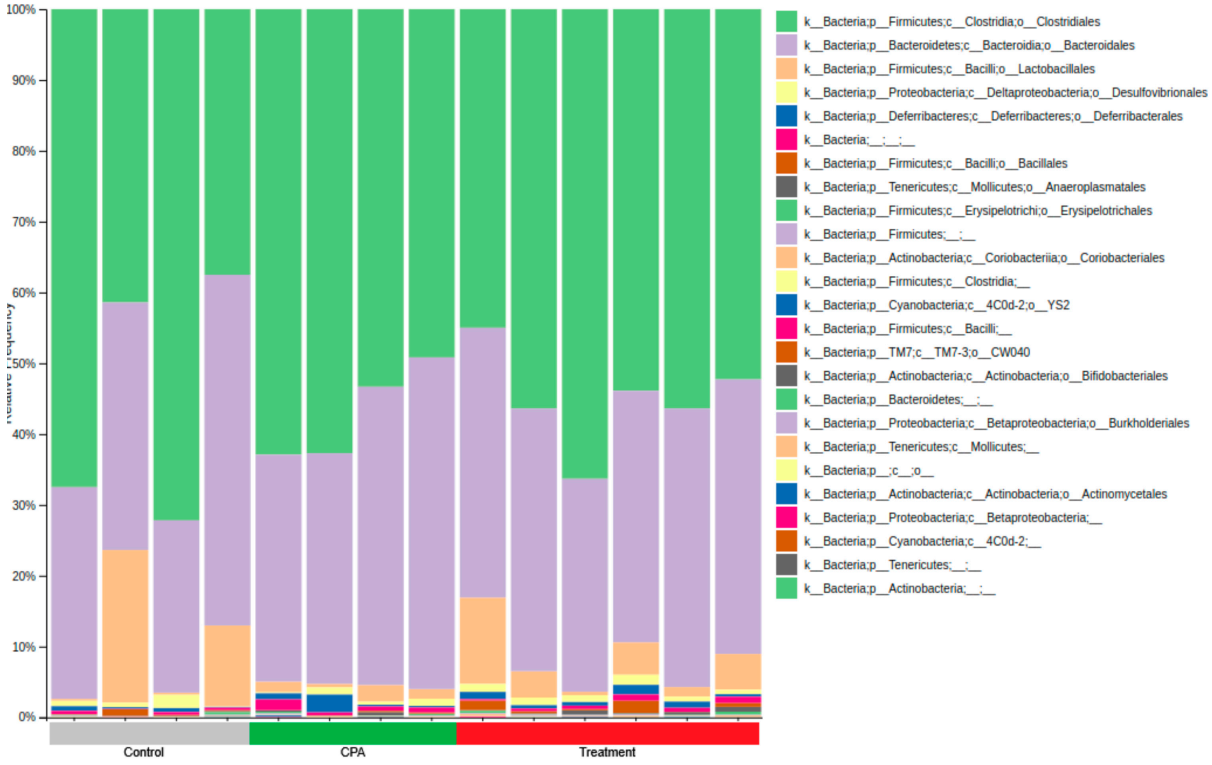

Figure S4. Bar plot showing the taxonomic distribution across all samples.

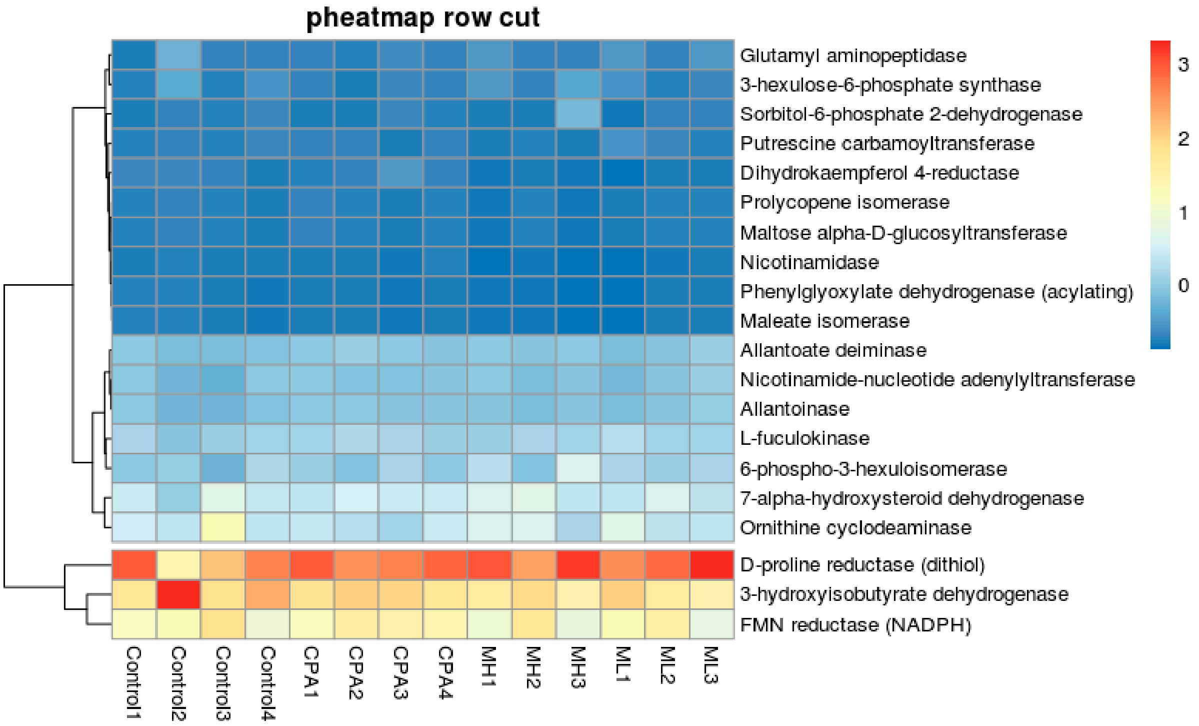

Figure S5. Heatmap showing the abundance of enzymes (EC numbers), which were differentially abundant in the 6MKF treatment.

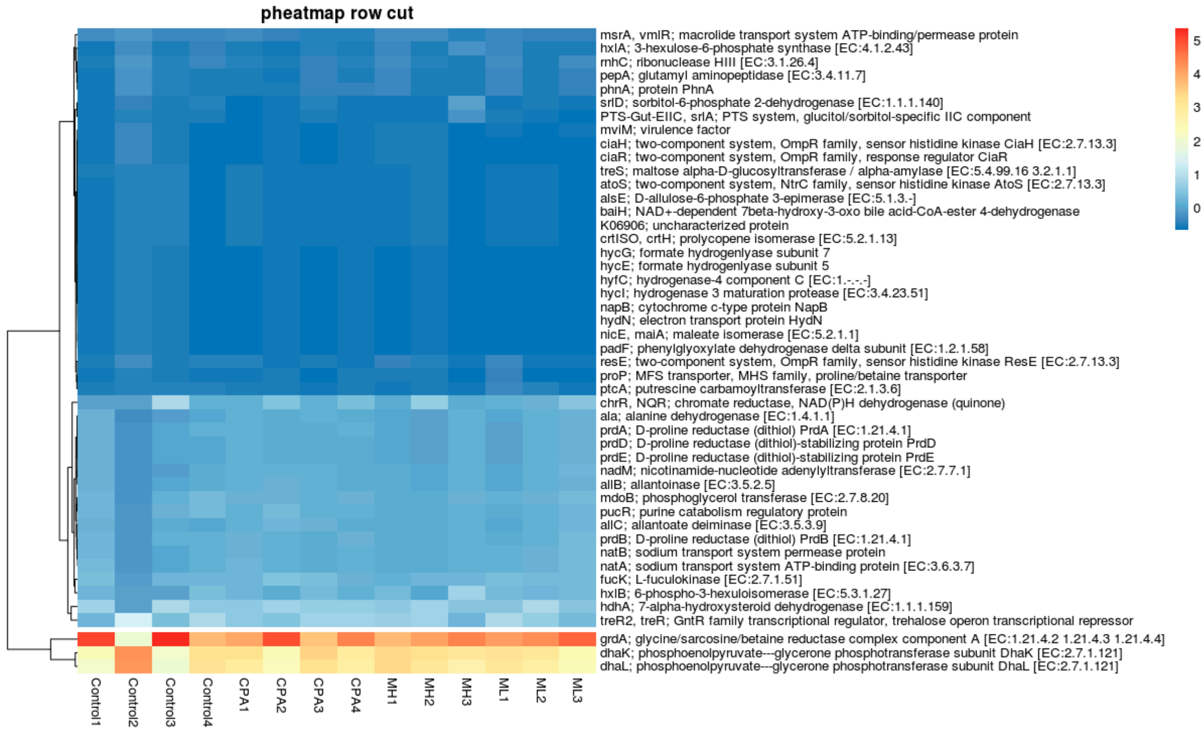

Figure S6. Heatmap showing the abundance of KO, which were differentially abundant in the 6MKF treatment.
